# Pyrroloquinoline Quinone Regulates Enteric Neurochemical Plasticity of Weaned Rats Challenged With Lipopolysaccharide

**DOI:** 10.3389/fnins.2022.878541

**Published:** 2022-05-03

**Authors:** Chenyu Shi, Song Xu, Caiyun Huang, Zijie Wang, Wenhui Wang, Dongxu Ming, Xindi Yin, Hu Liu, Fenglai Wang

**Affiliations:** ^1^State Key Lab of Animal Nutrition, College of Animal Science and Technology, China Agricultural University, Beijing, China; ^2^College of Animal Science, Fujian Agriculture and Forestry University, Fuzhou, China; ^3^Department of Nutrition and Health, China Agricultural University, Beijing, China

**Keywords:** pyrroloquinoline quinone, enteric nervous system, neurochemical plasticity, Akt signaling pathway, enteritis rats

## Abstract

The enteric nervous system (ENS) is important for the intestinal barrier to defend and regulate inflammation in the intestine. The aim of this study was to investigate the effect of pyrroloquinoline quinone (PQQ) on regulating neuropeptide secretion by ENS neurons of rats challenged with lipopolysaccharide (LPS) to create enteritis. Thirty Sprague Dawley rats were divided into five groups, namely, basal (CTRL), basal plus LPS challenge (LPS), basal with 2.5 mg/kg b.w./day of PQQ plus challenge with LPS (PQQ 2.5), basal with 5.0 mg/kg b.w./day PQQ plus challenge with LPS (PQQ 5), and basal with 10.0 mg/kg b.w./day PQQ plus challenge with LPS (PQQ 10). After treatment with basal diet or PQQ for 14 days, rats were challenged with LPS except for the CTRL group. Rats were euthanized 6 h after the LPS challenge. Rats showed an increased average daily gain in PQQ treatment groups (*P* < 0.05). Compared with the LPS group, PQQ 5 and PQQ 10 rats showed increased villus height and villus height/crypt depth of jejunum (*P* < 0.05). In PQQ treatment groups, concentrations of IL-1β and TNF-α in serum and intestine of rats were decreased, and IL-10 concentration was increased in serum compared with the LPS group (*P* < 0.05). Compared with the LPS group, the concentration of neuropeptide Y (NPY), nerve growth factor (NGF), vasoactive intestinal peptide (VIP), substance P (SP), calcitonin gene-related peptide (CGRP), and brain-derived neurotropic factor (BDNF) in serum were decreased in PQQ treatment groups (*P* < 0.05). Compared with the LPS group, ileal mRNA levels of BDNF, NPY, and NGF were decreased in PQQ treatment groups (*P* < 0.05). Jejunal concentrations of SP, CGRP, VIP, BDNF, NPY, and NGF were decreased in PQQ treatment groups compared with the LPS group (*P* < 0.05). Compared with the LPS group, phosphor-protein kinase B (p-Akt)/Akt levels in jejunum and colon were decreased in PQQ treatment groups (*P* < 0.05). In conclusion, daily treatment with PQQ improved daily gain, jejunal morphology, immune responses. PQQ-regulated enteric neurochemical plasticity of ENS *via* the Akt signaling pathway of weaned rats suffering from enteritis.

## Introduction

The intestine is the body organ with the largest membrane surface area that serves to absorb nutrients and sense, and recognize and defend against pathogens, antigens, toxins, and other detrimental secretions. The intestine accomplishes these diverse functions through coordinated effects of the enteroendocrine system, the enteric nervous system (ENS), the gut immune system, and the non-immune defense system of the intestine (Ferraris et al., 1989; De Giorgio, 2006). Intestinal nerve cells and glial cells in the ENS secrete neuropeptides and immune factors to pass signals between the ENS and the central nervous system (CNS). These signals influence mucosal secretions and gastrointestinal peristalsis and affect rhythms and hormone secretion in the CNS (Rao and Gershon, 2018; Boesmans et al., 2019). The ENS plays an important role in intestinal diseases, such as colitis, irritable bowel syndrome, and inflammatory bowel disease (Lasrado et al., 2017; Resnikoff et al., 2019; Jarret et al., 2020). The ENS, which includes submucosal nerve plexus, myenteric nerve plexus, and glial cells, usually is functional and structurally mature during the early life of mammals (Montedonico et al., 2006; Parathan et al., 2020). The capacity to regulate neuropeptides is called neurochemical plasticity. Enteric neuroplasticity is an adaption to intestinal contents and the intestinal microenvironment and is pronounced during early development in mammals (Moeser et al., 2017).

The phosphatidylinositol-3 kinase (PI3K)/protein kinase B (Akt) signaling pathway regulates the activity ofglycogen synthase kinase-3β (GSK-3β), Tau protein, and *N*-methyl-D-aspartame receptor (NMDA), which promotes the development of nerve cells and regeneration of synapses (Iqbal et al., 2016; Majewska and Szeliga, 2017; Falcon et al., 2019). The PI3K/Akt signaling pathway also plays an important regulatory role in intestinal neurological diseases by reducing the oxidative stress and apoptosis of nerve cells, improving the secretion of neurotropic factors, regulating survival and differentiation of nerve cells, and promoting the proliferation of intestinal nerve cells and glial cells (Ichihara et al., 2004; Du et al., 2009; Becker et al., 2013).

Pyrroloquinoline quinone (PQQ), a water-soluble quinone compound, was discovered as a redox cofactor of methanol dehydrogenase in pseudomonas TP1 and other Gram-negative bacteria (Duine et al., 1979; Duine, 1991). We demonstrated previously that dietary supplementation with PQQ could regulate intestinal morphology, mucosal barrier function, colonic microbiota, and antioxidant status to reduce diarrhea and improve the growth performance of weaned pigs (Yin et al., 2019; Huang et al., 2020, 2021; Ming et al., 2021). PQQ is a potent neuroprotective nutrient in the CNS and peripheral nerves, which can mitigate sciatic nerve injury, reduce neurotoxin-induced neurotoxicity, and promote neuronal cell regeneration (Liu et al., 2005; Hara et al., 2007; Shanan et al., 2019). PQQ treatment can ameliorate signs of memory impairment in aging mice and schizophrenic rats *via* reducing the expression of phosphorylation Akt and maintaining the GSK-3β level in the hippocampus (Zhou X. Q. et al., 2018; Zhou et al., 2020).

We hypothesized that PQQ treatment could influence the ENS and regulate enteric neuroplasticity to improve intestinal health. We tested this hypothesis in the enteritis rat model *via* developed challenging rats with lipopolysaccharide (LPS). We detected neuropeptides in serum and intestine and Akt signaling pathway expression in the intestine to determine if PQQ could regulate enteric neuroplasticity *via* the Akt signaling pathway.

## Materials and Methods

### Animals and Experimental Treatment

All experimental protocols in this study were approved by the Animal Subjects Committee of China Agricultural University (Beijing, China) and carried out based on the National Research Council’s Guide for the Care and Use of Laboratory Animals (AW01211202-1-2). Sprague Dawley male rats (*n* = 30, 21 days old) were purchased from SPF (Beijing) Biotechnology Co. Ltd., and were housed in a pathogen-free animal room with a 12/12 h light-dark cycle at 23°C. After 3 days of acclimatization, rats were divided randomly into 5 groups (*n* = 6 per treatment group), namely, (1) basal unchallenged (CTRL), (2) LPS challenged (LPS), (3) 2.5 mg/kg b.w./day low dose of PQQ and challenged with LPS (PQQ 2.5), (4) 5.0 mg/kg b.w./day medium dose of PQQ and challenged with LPS (PQQ 5), and (5) 10.0 mg/kg b.w./day high dose of PQQ and challenged with LPS (PQQ 10) (Lu et al., 2015). PQQ⋅Na_2_ (purity, ≥98%; Changmao Biochemical Engineering Co. Ltd., Changzhou, China) dissolved in physiological saline was administrated intragastrically for 14 days. All rats were weighed every day and had free access to water and food. After the supplementation period, rats were injected with LPS [4 mg/kg b.w., isolated from *Escherichia coli* (serotype 055: B5) (Zani et al., 2008), purchased from Sigma, United States] except rats in the CTRL group on day 15. All rats were euthanized by intraperitoneal injection with pentobarbitone sodium (50 mg/kg b.w.) (Mohamed et al., 2020) at 6 h after the LPS dose.

### Sample Collection

After euthanasia, serum samples were collected from the abdominal veins of rats and separated into serum and stored at −20°C for further analysis. Jejunal and colonic tissues (1 cm^2^) were excised and stored in 4% paraformaldehyde solution (40% formaldehyde solution dissolved in phosphate-buffered saline (PBS)) for analysis of intestinal morphology. Notably, 2 cm segments from the middle of the jejunum, ileum, and colon were collected in freezing tubes which were frozen rapidly in liquid nitrogen and stored at −80°C for further analysis.

### Jejunal Morphology

After fixation with 4% paraformaldehyde solution for 24 h, tissue samples were dehydrated and embedded in paraffin. Jejunal sections (5 μm) were stained with hematoxylin and eosin. Villus height (VH), crypt depth (CD), and villus height/crypt depth ratio (VCR) were viewed and evaluated using a microscope (Eclipse CI, Nikon) and imaging software (DS-U3, Nikon). Data were collected from at least 10 well-oriented villi and crypts from 5 slides per sample.

### Cytokines of Serum and Intestinal Segments

Cytokines, including interleukin (IL)-1β, IL-6, IL-10, and tumor necrosis factor (TNF)-α, were determined in serum, jejunum, ileum, and colon using enzyme-linked immunoassay (ELISA) kits (Beijing Kang Iia Hong Yuan Biological Technology Co., Ltd., Beijing, China) according to the manufacturer’s instructions and quantified using a Multiskan Microplate Reader (Thermo Fisher Scientific, United States). Absorbance for kits was all set at 450 nm, and the minimal detections were 31.25 pg/ml for IL-1β and IL-6, 15.63 pg/ml for IL-10, and 9.38 pg/ml for TNF-α. The intra- and inter-assay coefficients of variation (CV) were <10% for each assay.

### Quantitative Real-Time Polymerase Chain Reaction

Total RNA was extracted from the snap-frozen ileal tissue using RNAiso Plus (9109, TaKaRa Bio, Inc., Japan)/chloroform extraction. Complementary DNA (cDNA) was synthesized using the PrimeScript RT Reagent Kit with gDNA Eraser (RR047A, TaKaRa Bio, Inc., Japan). Quantitative real-time polymerase chain reaction PCR (RT-PCR) was conducted using the Roche Light Cycler^®^ System (Roche, South San Francisco, CA, Canada). The primer sequences are shown in Supplementary Table 1. Target genes were detected by normalizing with β-actin and calculating using the 2^–ΔΔ*CT*^ method (Pfaffl et al., 2002).

### Determination of the Concentration of Neuropeptides

Neuropeptides including neuropeptide Y (NPY), nerve growth factor (NGF), vasoactive intestinal peptide (VIP), substance P (SP), calcitonin gene-related peptide (CGRP), and brain-derived neurotropic factor (BDNF) were determined in serum using assay kits according to the manufacturer’s instructions (Supplementary Table 2). These same neuropeptides were determined in the jejunum, ileum, and colon.

### Immunohistochemistry

Fixed jejunum and colon tissues were embedded in paraffin and cut into sections (5 μm) followed by deparaffinization and rehydration. Tissue sections were placed in ethylenediaminetetraacetic acid (EDTA) antigen retrieval buffer (pH 8.0) to retrieve the antigen. Endogenous peroxidase activity was blocked with 3% H_2_O_2_ under dark conditions at room temperature and was then washed with PBS (PBS, pH 7.4). Bovine serum albumin (BSA, 5%) was added to tissues. Primary antibodies (Supplementary Table 3) diluted in PBS were incubated with tissue sections overnight at 4°. Tissue sections were covered with secondary antibodies for 50 min at room temperature, and positive expression for protein gene product 9.5 (PGP9.5), SP, CGRP, BDNF, NPY, and NGF was dyed brown with diaminobenzidine (DAB). Hematoxylin was used as a counterstain for nuclei dyed blue. Ganglia immunoreactive for neuropeptides was calculated in percentage in the total area of neurons.

### Western Blot Assay

Jejunal and colonic tissues were ground in liquid nitrogen and lysed using radioimmunoprecipitation assay (RIPA) buffer with protease and phosphatase inhibitors. After sonication and centrifugation, protein concentration in the supernatant was quantified using a bicinchoninic acid (BCA) protein assay kit (02912E, CWbiotech, Beijing, China). Supernatant with 30 μg proteins from each sample was separated in sodium dodecyl sulfate (SDS) polyacrylamide gels and then transferred to polyvinylidene fluoride (PVDF) membranes (0.45 μm, Millipore, United States). Membranes were blocked and incubated with the primary antibodies of phosphatidylinositol 3-kinase (PI3K, #4257, Cell Signaling Technology, Danvers, MA, United States), protein kinase B (Akt, #9272, Cell Signaling Technology), phosphor-Akt (p-Akt, #9271, Cell Signaling Technology), and β-actin (#4970, Cell Signaling Technology) overnight at 4°C. The membranes were incubated with secondary antibodies (111-035-003, Jackson, United States) for 1 h at room temperature and reacted with electrochemiluminescence (ECL, WBKLS0500, Millipore, United States). The intensity of protein bands was analyzed using the ImageJ software.

### Statistical Analysis

All data were analyzed with a one-way analysis of variance (ANOVA) using SAS (version 9.2, United States). Differences among mean values were evaluated using the Duncan’s multiple range test. The individual rat was the experimental unit for traits of interest. Values are expressed as means and considered statistically different if *P* ≤ 0.05. Figures were created using GraphPad Prism 9.

## Results

### Average Daily Gain

**FIGURE 1:**
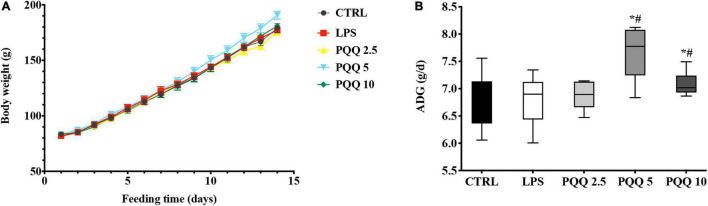
Average daily gain of weaned rats (*n* = 6). **(A)** Body weight of rats was recorded with feeding time. **(B)** Average daily gain(ADG) of rats was evaluated by body weight. CTRL, control treatment; lipopolysaccharide (LPS), control and LPS treatment; pyrroloquinoline quinone (PQQ) 2.5, intragastric administration with 2.5 mg/kg b.w./day PQQ⋅Na_2_ treatment; PQQ 5, intragastric administration with 5.0 mg/kg b.w./day PQQ⋅Na_2_treatment; PQQ 10, intragastric administration with 10.0 mg/kg b.w./day PQQ⋅Na_2_ treatment. *Means significant difference with the CTRL group (*P* ≤ 0.05); ^#^means significant difference with the LPS group (*P* ≤ 0.05).

Compared with CTRL and LPS groups, rats assigned to PQQ 5 and PQQ 10 groups expressed increased average daily gain (ADG) (*P* < 0.05, Figure 1). Rats assigned to the PQQ 5 group showed the greatest difference in ADG compared with both CTRL and LPS groups.

### Jejunal Morphology

**FIGURE 2:**
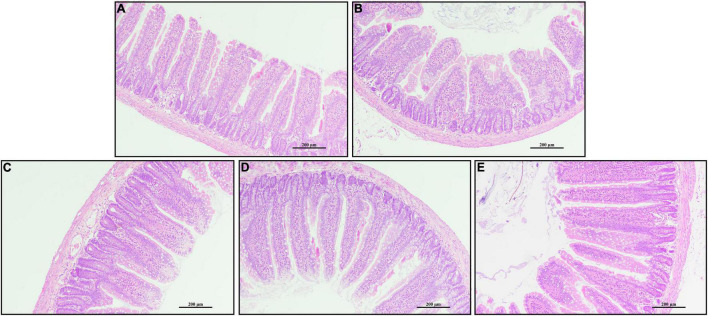
Jejunal morphology by H and E stains. **(A)** Control treatment; **(B)** control and LPStreatment; **(C)** intragastric administration with 2.5 mg/kg b.w./day PQQ⋅Na_2_ treatment; **(D)** intragastric administration with 5.0 mg/kg b.w./day PQQ⋅Na_2_ treatment; and **(E)** intragastric administration with 10.0 mg/kg b.w./day PQQ⋅Na_2_ treatment. Scale bars in pictures are 200 μm.

Rats challenged with LPS had non-distinct jejunal villus compared with the CTRL group (Figure 2). Rats in PQQ 2.5, PQQ 5, and PQQ 10 groups exhibited more complete and taller jejunal villus compared with the LPS group. Compared with the CTRL group, LPS treatment decreased jejunal VH and VCR in rats (*P* < 0.05, Table 1). Compared with the LPS group, PQQ 5 and PQQ 10 groups increased VH and VCR of the jejunum (*P* < 0.05). Neither LPS nor PQQ treatments affected CD.

**TABLE 1:** Jejunal villous morphology of weaned rats (*n* = 6).^1^

Items	Treatments						
						SEM	*P*-values
	CTRL	LPS	PQQ 2.5	PQQ 5	PQQ 10		
VH, μm	434.20	373.44*	389.78	472.57^#^	436.88^#^	10.99	0.02
CD, μm	113.79	120.79	121.82	122.60	112.33	2.52	0.60
VCR	3.86	3.12*	3.23*	3.92^#^	3.92^#^	0.11	0.03

^1^CTRL, control treatment; LPS, control and LPS treatment; pyrroloquinoline quinone (PQQ) 2.5, intragastric administration with 2.5 mg/kg b.w./day PQQ⋅Na_2_ treatment; PQQ 5, intragastric administration with 5.0 mg/kg b.w./day PQQ⋅Na_2_ treatment; PQQ 10, intragastric administration with 10.0 mg/kg b.w./day PQQ⋅Na_2_ treatment. VH, villus height; CD, crypt depth; VCR, villus height/crypt depth. *Means significant difference with the CTRL group (P ≤ 0.05); ^#^means significantdifference with the LPS group (P ≤ 0.05).

### Cytokines in Serum and Intestine

**FIGURE 3:**
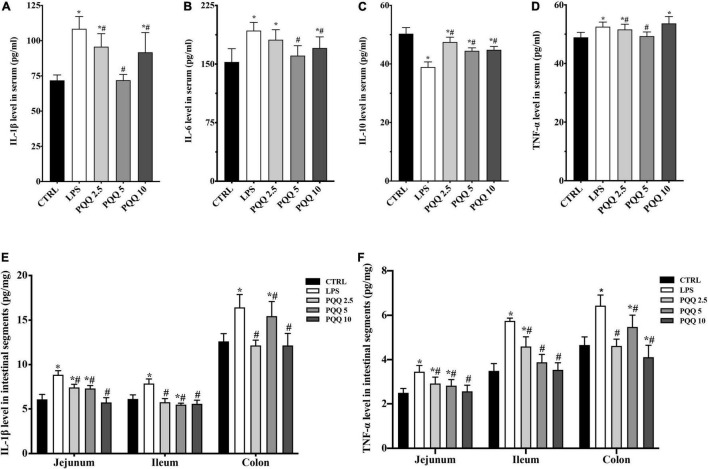
Cytokine level in serum and intestinal segments of weaned rats. **(A)** IL-1β level in serum (*n* = 6); **(B)** IL-6 level in serum (*n* = 6); **(C)** IL-10 level in serum (*n* = 5); **(D)** TNF-α level in serum (*n* = 6); **(E)** IL-1β level in jejunum, ileum and colon (*n* = 6); and **(F)** TNF-α level in jejunum, ileum and colon (*n* = 6). CTRL, control treatment; LPS, control and LPS treatment; PQQ 2.5, intragastric administration with 2.5 mg/kg b.w./day PQQ⋅Na_2_ treatment; PQQ 5, intragastric administration with 5.0 mg/kg b.w./day PQQ⋅Na_2_ treatment; PQQ 10, intragastric administration with 10.0 mg/kg b.w./day PQQ⋅Na_2_ treatment. *Means significant difference with the CTRL group (*P* ≤ 0.05); ^#^means significant difference with the LPS group (*P* ≤ 0.05).

Compared with the CTRL group, concentrations of IL-1β, IL-6, and TNF-α increased, and IL-10 decreased in the serum of rats challenged with LPS (*P* < 0.05, Figure 3). Feeding PQQ at 2.5, 5.0, and 10.0 mg/kg b.w. to rats decreased (*P* < 0.05) concentrations of IL-1β and TNF-α, and increased concentration of IL-10 in serum compared with the LPS group. With a similar pattern, concentrations of IL-1β and TNF-α in jejunum, ileum, and colon were increased (*P* < 0.05) in the LPS group compared with the CTRL group. Concentrations of IL-1β and TNF-α in jejunum, ileum, and colon of rats were decreased (*P* < 0.05)compared with rats assigned to the LPS group.

### Concentration of Neuropeptides in Serum

**FIGURE 4:**
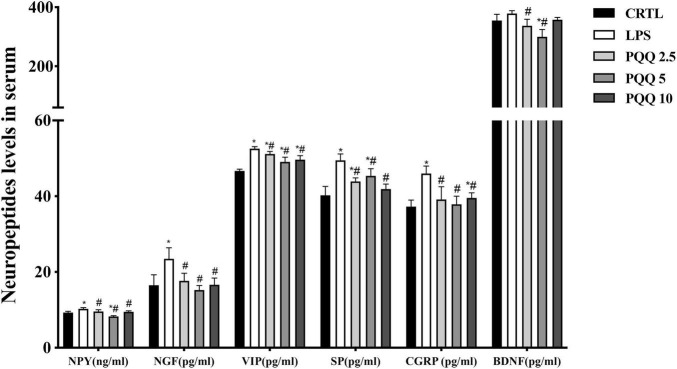
Ileal neuropeptide mRNA expression levels of weaned rats (*n* = 6). SP, substance P; CGRP, calcitonin gene-related peptide; BDNF, brain-derived neurotropic factor; NPY, neuropeptide Y; NGF, nerve growth factor. CTRL, control treatment; LPS, control and LPS treatment; PQQ 2.5, intragastric administration with 2.5 mg/kg b.w./day PQQ⋅Na_2_ treatment; PQQ 5, intragastric administration with 5.0 mg/kg b.w./day PQQ⋅Na_2_ treatment; PQQ 10, intragastric administration with 10.0 mg/kg b.w./day PQQ⋅Na_2_ treatment. *Means significant difference with the CTRL group (*P* ≤ 0.05); ^#^means significant difference with the LPS group (*P* ≤ 0.05).

Compared with the CTRL group, concentrations of NPY, NGF, VIP, SP, and CGRP were increased (*P* < 0.05, Figure 4) in the serum of rats in the LPS group. There were no significant differences in BDNF between LPS and CTRL groups. Concentrations of NPY, NGF, VIP, SP, CGRP, and BDNF were decreased (*P* < 0.05) with PQQ intake compared with the LPS group. Feeding PQQ at 5.0 mg/kg b.w. decreased concentrations of NPY, NGF, VIP, and CGRP more than 2.5 or 10.0 mg/kg b.w. when compared with the LPS group.

### mRNA Abundance of Neuropeptides in the Ileum

**FIGURE 5:**
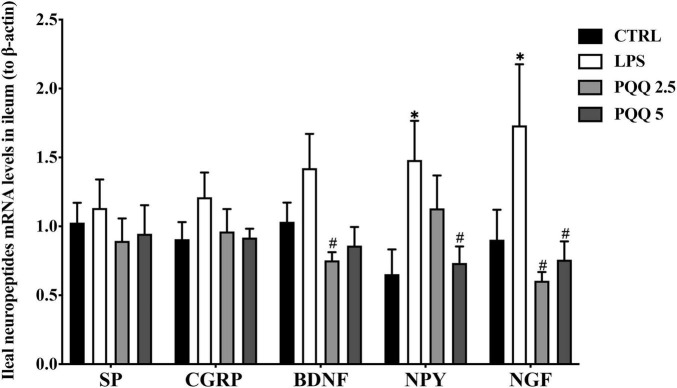
Neuropeptide levels in serum of weaned rats. NPY, neuropeptide Y (*n* = 6); NGF, nerve growth factor (*n* = 5); VIP, vasoactive intestinal peptide (*n* = 6); SP, substance P (*n* = 5); CGRP, calcitonin gene-related peptide (*n* = 5); BDNF, brain-derived neurotropic factor (*n* = 5). CTRL, control treatment; LPS, control and LPS treatment; PQQ 2.5, intragastric administration with 2.5 mg/kg b.w./day PQQ⋅Na_2_ treatment; PQQ 5, intragastric administration with 5.0 mg/kg b.w./day PQQ⋅Na_2_ treatment; PQQ 10, intragastric administration with 10.0 mg/kg b.w./day PQQ⋅Na_2_ treatment. *Means significant difference with the CTRL group (*P* ≤ 0.05); ^#^means significant difference with the LPS group (*P* ≤ 0.05).

Compared with the CTRL group, mRNA concentrations for NPY and NGF were increased (*P* < 0.05, Figure 5) in the LPS group. Compared with the LPS group, mRNA concentrations for BDNF and NGF were decreased (*P* < 0.05) in PQQ 2.5, and mRNA concentration for NPY decreased (*P* < 0.05) in PQQ 5.

### Concentration of Neuropeptides in Jejunum, Ileum and Colon

Compared with the CTRL group, concentrations of all detected neuropeptides were increased (*P* < 0.05, Table 2) in the jejunum and ileum of rats in the LPS group. Compared with the LPS group, concentrations of CGRP, VIP, NPY, and NGF were decreased (*P* < 0.05) in PQQ-fed groups in the jejunum, and SP and BDNF concentrations were decreased (*P* < 0.05) in PQQ 5 and PQQ 10 groups. In the ileum, concentrations of all neuropeptides measured were decreased (*P* < 0.05) in all PQQ-fed groups. In the colon, VIP concentration was increased, and BDNF concentration was decreased in the LPS group compared with the CTRL group (*P* < 0.05). Compared with the LPS group, colonic SP concentration was decreased (*P* < 0.05) in PQQ 10, CGRP concentration was increased (*P* < 0.05) in PQQ 5, VIP concentration was decreased (*P* < 0.05) in PQQ 2.5 and PQQ 5, BDNF concentration was increased (*P* < 0.05) in PQQ 5, NPY concentration was increased (*P* < 0.05) in PQQ 10, and NGF concentration was increased in PQQ 5.

**TABLE 2:** Neuropeptide concentration in intestinal segments of weaned rats (*n* = 6).^1^

Items	Treatments						
						SEM	*P*-values
	CTRL	LPS	PQQ 2.5	PQQ 5	PQQ 10		
**Jejunum**							
SP (pg/mg)	5.98	8.10*	7.30*	6.28^#^	5.95^#^	0.32	< 0.01
CGRP (pg/mg)	8.53	11.02*	10.22*^#^	8.63^#^	8.68^#^	0.24	< 0.01
VIP (pg/mg)	56.56	82.00*	53.60^#^	65.55^#^	46.89^#^	4.47	< 0.01
BDNF (pg/mg)	9.84	14.80*	13.03*	10.61^#^	9.88^#^	0.92	0.01
NPY (ng/mg)	13.14	17.58*	10.96^#^	13.07^#^	10.32*^#^	0.77	< 0.01
NGF (pg/mg)	6.15	9.77*	7.24*^#^	6.33^#^	6.05^#^	0.17	< 0.01
**Ileum**							
SP (pg/mg)	4.96	7.82*	5.03^#^	5.07^#^	5.35*^#^	0.10	< 0.01
CGRP (pg/mg)	14.62	19.34*	15.63^#^	14.42^#^	15.87^#^	0.45	< 0.01
VIP (pg/mg)	31.44	44.58*	27.72*^#^	32.09^#^	34.42*^#^	0.98	< 0.01
BDNF (pg/mg)	27.69	42.70*	32.01*^#^	35.43*^#^	34.83*^#^	0.95	< 0.01
NPY (ng/mg)	6.62	8.53*	6.01*^#^	6.42^#^	7.31*^#^	0.20	< 0.01
NGF (pg/mg)	4.72	7.83*	5.00^#^	4.43^#^	5.99*^#^	0.19	< 0.01
**Colon**							
SP (pg/mg)	14.61	15.33	15.21	16.66	12.30*^#^	0.64	0.01
CGRP (pg/mg)	19.17	18.09	18.45	22.41*^#^	16.74	0.86	< 0.01
VIP (pg/mg)	71.69	103.69*	87.62*^#^	72.60^#^	100.42*	4.43	< 0.01
BDNF (pg/mg)	74.59	38.75*	39.55*	63.84^#^	50.57*	3.75	< 0.01
NPY (ng/mg)	15.3	13.86	15.55	13.28*	20.73*^#^	0.68	< 0.01
NGF (pg/mg)	12.98	11.85	11.11*	14.24^#^	11.88	0.68	0.03

*^1^SP, substance P; CGRP, calcitonin gene-related peptide; VIP, vasoactive intestinal peptide; BDNF, brain-derived neurotropic factor; NPY, neuropeptide Y; NGF, nerve growth factor. CTRL, control treatment; lipopolysaccharide (LPS), control and LPS treatment; PQQ 2.5, intragastric administration with 2.5 mg/kg b.w./day PQQ⋅Na_2_ treatment; PQQ 5, intragastric administration with 5.0 mg/kg b.w./day PQQ⋅Na_2_ treatment; PQQ 10, intragastric administration with 10.0 mg/kg b.w./day PQQ⋅Na_2_ treatment.*

*Means significant difference with the CTRL group (P ≤ 0.05); ^#^means significantdifference with the LPS group (P ≤ 0.05).

### Immunostaining of Neuropeptides in Jejunum and Colon

**FIGURE 6:**
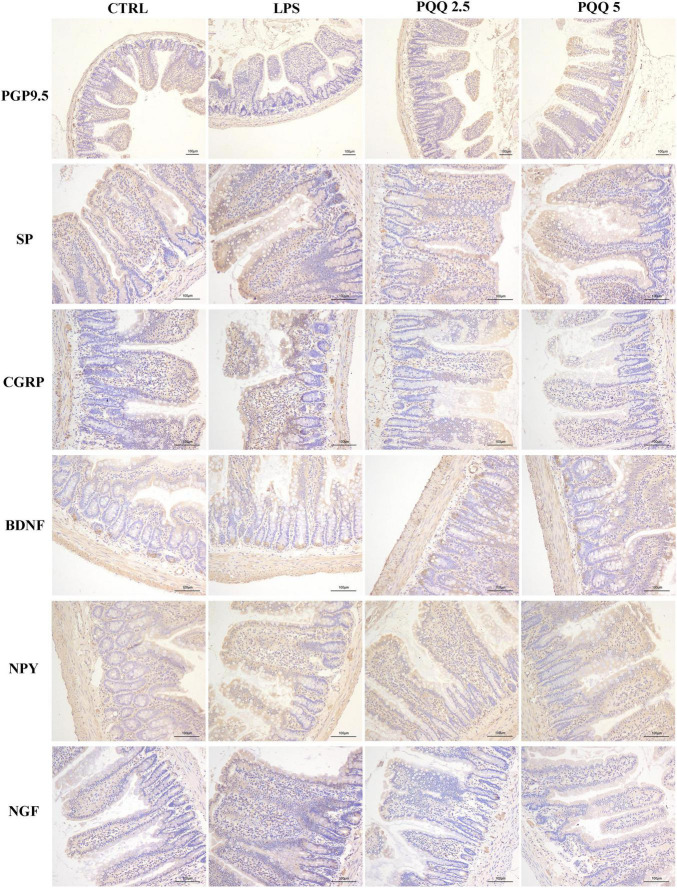
Immunohistochemical staining of jejunal neurons (*n* = 6). PGP9.5, protein gene product 9.5; SP, substance P; CGRP, calcitonin gene-related peptide; BDNF, brain-derived neurotropic factor; NPY, neuropeptide Y; NGF, nerve growth factor. CTRL, control treatment; LPS, control and LPS treatment; PQQ 2.5, intragastric administration with 2.5 mg/kg b.w./day PQQ⋅Na_2_ treatment; PQQ 5, intragastric administration with 5.0 mg/kg b.w./day PQQ⋅Na_2_ treatment; PQQ 10, intragastric administration with 10.0 mg/kg b.w./day PQQ⋅Na_2_ treatment. Scale bar = 100 μm.

Compared with the CTRL group, the percentage of ganglia immunoreactive for PGP9.5 in the jejunum was decreased (*P* < 0.05, Table 3) in the LPS group and showed a lighter PGP9.5-positive area dyed with brown (Figure 6). Compared with the LPS group, the PGP9.5 immunoreactive percentage of ganglia in the jejunum of rats was increased (*P* < 0.05, Table 3) in the PQQ 5 group. Compared with the CTRL group, NGF-positive surface area in the jejunum was increased (*P* < 0.05, Table 3) and dyed darker (Figure 6) in the LPS group.

**TABLE 3:** Percentage of ganglia immunoreactive for neuropeptides in the total area by immunohistochemical staining (*n* = 6, %).^1^

Items	Treatments					
					SEM	*P*-values
	CTRL	LPS	PQQ 2.5	PQQ 5		
**Jejunum**						
PGP9.5	16.88	6.70*	10.79	16.80^#^	1.47	0.03
SP	0.30	0.43	0.29	0.26	0.03	0.26
CGRP	0.10	0.20	0.09	0.19	0.02	0.25
BDNF	0.49	0.37	0.38	0.32	0.03	0.23
NPY	0.30	0.45	0.59	0.28	0.06	0.26
NGF	2.83	4.11*	4.41*	4.00*	0.19	0.01
**Colon**						
PGP9.5	20.06	16.42	16.38	24.77	2.07	0.46
SP	0.06	0.14*	0.04^#^	0.07^#^	0.01	0.00
CGRP	0.09	0.08	0.10	0.18^#^	0.02	0.23
BDNF	0.77	0.40	0.42	0.52	0.08	0.42
NPY	0.44	0.36	0.28	0.61^#^	0.05	0.05
NGF	3.45	2.48	3.16	5.19^#^	0.41	0.13

*^1^PGP9.5, protein gene product 9.5; CGRP, calcitonin gene-related peptide; NGF, nerve growth factor. CTRL, control treatment; LPS, control and LPS treatment; PQQ 2.5, intragastric administration with 2.5 mg/kg b.w./day PQQ⋅Na_2_ treatment; PQQ 5, intragastric administration with 5.0 mg/kg b.w./day PQQ⋅Na_2_ treatment; PQQ 10, intragastric administration with 10.0 mg/kg b.w./day PQQ⋅Na_2_ treatment.*

*Means significant difference with the CTRL group (P ≤ 0.05); ^#^means significantdifference with the LPS group (P ≤ 0.05).

**FIGURE 7:**
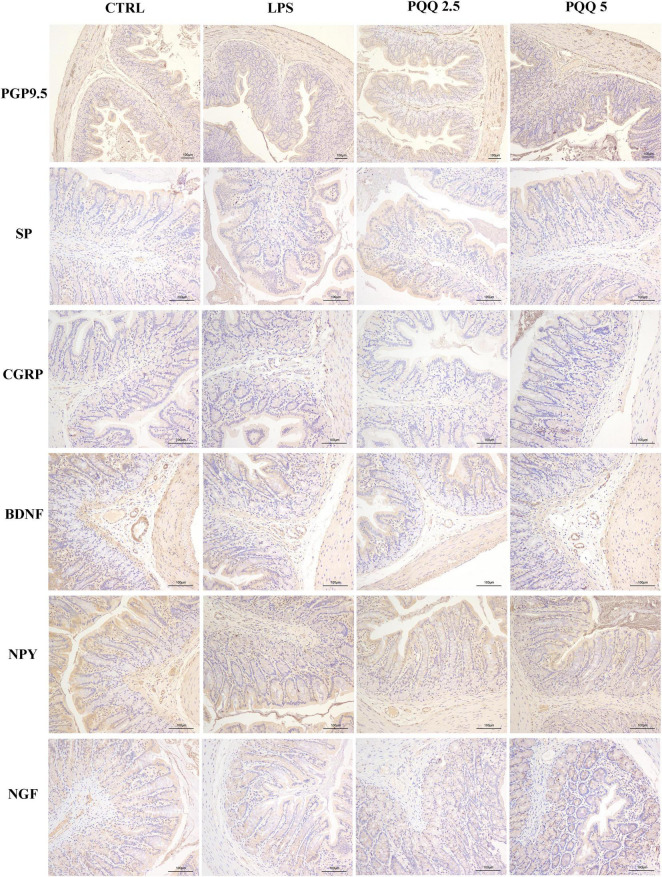
Immunohistochemical staining of colonic neurons (*n* = 6). PGP9.5, protein gene product 9.5; SP, substance P; CGRP, calcitonin gene-related peptide; BDNF, brain-derived neurotropic factor; NPY, neuropeptide Y; NGF, nerve growth factor. CTRL, control treatment; LPS, control and LPS treatment; PQQ 2.5, intragastric administration with 2.5 mg/kg b.w./day PQQ⋅Na_2_ treatment; PQQ 5, intragastric administration with 5.0 mg/kg b.w./day PQQ⋅Na_2_ treatment; PQQ 10, intragastric administration with 10.0 mg/kg b.w./day PQQ⋅Na_2_ treatment. Scale bar = 100 μm.

In the colon, SP-positive surface area increased (*P* < 0.05, Table 3) in the LPS group compared with the CTRL group. PQQ treatments decreased (*P* < 0.05, Table 3) SP-positive surface area in the colon compared with the LPS group. Colonic CGRP, NPY, and NGF were increased in the PQQ 5 group compared with the LPS group (*P* < 0.05, Table 3) and dyed darker (Figure 7) in the PQQ 5 group.

### Activation of the Akt Pathway in Jejunum and Colon

**FIGURE 8:**
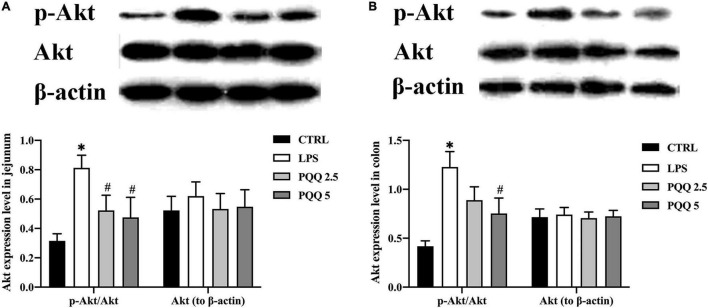
Abundance of the Akt pathway in jejunal and colonic tissues of weaned rats (*n* = 6). **(A)** p-Akt, Akt, and PI3K abundances to β-actin in jejunum; **(B)** p-Akt, Akt, andPI3K abundances to β-actin in the colon. PI3K, phosphatidylinositol-3 kinase; Akt, protein kinase B; CTRL, control treatment; LPS, control and LPS treatment; PQQ 2.5, intragastric administration with 2.5 mg/kg b.w./day PQQ⋅Na_2_ treatment; PQQ 5, intragastric administration with 5.0 mg/kg b.w./day PQQ⋅Na_2_ treatment; PQQ 10, intragastric administration with 10.0 mg/kg b.w./day PQQ⋅Na_2_ treatment. *Means significant difference with the CTRL group (*P* ≤ 0.05); ^#^means significant difference with the LPS group (*P* ≤ 0.05).

Compared with the CTRL group, p-Akt/Akt was increased in LPS groups both in the jejunum and colon ofrats (*P* < 0.05, Figures 8A,B). Compared with the LPS group, p-Akt/Akt was decreased (*P* < 0.05, Figure 8A) in the jejunum of rats both in PQQ 2.5 and PQQ 5 groups. Additionally, p-Akt/Akt was decreased in the colon of rats in the PQQ 5 group compared with the LPS group (*P* < 0.05, Figure 8B). The abundance of PI3K was not affected by any treatments in both the jejunum and colon.

## Discussion

Pyrroloquinoline quinone is a natural antioxidant that has been used in the treatment of osteoporosis, muscle atrophy, radiation poisoning, and arthritis and promotes the growth of the organisms by regulating oxidation, repairing DNA damage, reducing apoptosis, and maintaining mitochondrial function (Kasahara and Kato, 2003; Huang et al., 2017; Wu et al., 2017; Xu et al., 2018; Geng et al., 2019; Jiang et al., 2019). We previously demonstrated that dietary PQQ could increase ADG and gain to feed ratio and reduce diarrhea incidence in weaned pigs (Yin et al., 2019; Huang et al., 2021; Ming et al., 2021). In this study, daily treatment with 5.0 mg/kg and 10.0 mg/kg PQQ increased ADG of rats compared with rats not supplemented with PQQ.

We developed the enteritis model by challenging rats with LPS and verified the existence of enteritis by evaluating jejunal morphology and cytokines in serum and intestine. LPS is a component of Gram-negative bacteria that can damage the intestinal barrier cause immune dysfunction, initiate apoptosis of intestine epithelial cells, and develop enteritis (Yoshioka et al., 2009; Li et al., 2018; Dong et al., 2020). In this study, rats challenged with LPS developed inflamed intestines as evidenced by damaged jejunal morphology, increased concentrations of IL-1β, IL-6, and TNF-α in serum and IL-1β and TNF-α in intestine, and decreased IL-10 in serum. These observations confirmed that rats challenged with LPS developed intestinal inflammation.

Intestinal morphology and immune function improved with PQQ supplementation of LPS-challenged rats. Intestinal damage reduces absorption and metabolism of nutrients and compromises the integrity of the intestinal barrier (Baumgart and Carding, 2007; Turner, 2009; Wang et al., 2015; Zhou et al., 2018). Intestinal morphology can be improved in weaned rats when they were supplemented with PQQ (Zhang et al., 2019). Our previous study showed that dietary supplementation with PQQ can increase intestinal VH and VCR in weaned pigs (Yin et al., 2019). In this study, PQQ treatment increased jejunal VH and VCR compared with rats challenged with LPS. PQQ has anti-inflammatory effects and can reduce arthritis by inhibiting the production of pro-inflammatory cytokines such as TNF-α and IL-6 (Harris et al., 2013; Liu et al., 2016). In our previous study, PQQ supplementation reduced gut inflammation which improved intestinal health and growth of weaned pigs (Yin et al., 2019; Huang et al., 2020). In this study, PQQ supplementation decreased concentrations of pro-inflammatory cytokines in serum and intestine and increased concentrations of the anti-inflammatory cytokine and IL-10 in serum, which confirmed that PQQ regulates immune responses.

Pyrroloquinoline quinone can regulate enteric nervous damage induced by LPS challenge. The ENS regulates intestinal secretions, peristalsis, and immunity through endocrine substances and neuropeptides secreted by myenteric neurons, submucosal neurons, and glial cells (Rao and Gershon, 2018; Boesmans et al., 2019). Normally, the neuropeptides are divided into inhibitory neurotransmitters and excitatory neurotransmitters to control intestinal relaxation and contraction, respectively (Rao and Gershon, 2018). When the intestine suffers pressure, mucosal damage, and inflammation, the ENS can release neuropeptides to regulate intestinal peristaltic and immune factors (Jakob et al., 2020). PGP 9.5 is a neuroendocrine marker and is reduced with intestinal inflammation and damage (Resnikoff et al., 2019; Heymans et al., 2020). In this study, the immunoreactive percentage of PGP9.5 in the jejunum was decreased by LPS treatment and increased by PQQ treatment, suggesting that PQQ reduced the damage of jejunal neurons caused by LPS.

Enteric neurochemical plasticity was regulated with PQQ treatment by regulating immunoreactive neurons and concentrations of neuropeptides. Neurochemical plasticity is the variable or modifiable changes of the nervous system elicited by adaptation to the environment. During the early development of rats, the ENS matures gradually and has the strongest neurochemical plasticity (Rza̧p et al., 2020). As the first isolated neuropeptide and known as the prototypic tachykinin (TK), SP stimulates systemic pain and vasodilation, regulates immunity, promotes contraction as an excitatory neurotransmitter, and inhibits the secretion of digestive juices in the intestine (Euler and Gaddum, 1931; Holzer, 1998; Engel et al., 2012; Nässel et al., 2019). The concentration of SP is enhanced in the blood of rats suffering from colitis (Holzer, 1998). As a systemic vasodilator, CGRP simulates pain and regulates intestinal immunity similar to SP (Holzer, 1998; Engel et al., 2012; Lai et al., 2020). Sensory neurons in the gut of mice release CGRP to defend against *Salmonella* infection, and neurogenic inflammation challenged with LPS can promote CGRP release *via* activation of Toll-like receptor 4 (TLR4) in sensory neurons (Meseguer et al., 2014; Lai et al., 2020). As an inhibitory neurotransmitter and neuroprotective agent in the intestine, VIP is also an immunomodulator (Gonzalez-Rey and Delgado, 2005; Arciszewski et al., 2008). Rat myenteric neurons challenged with LPS increase the expression of VIP (Arciszewski et al., 2008). Except for promoting differentiation, development, and survival of neurons, BDNF regulates the sensitivity of the colon and rectum to constipation (Delafoy et al., 2006). It is synthesized by sensory neurons to mediate inflammatory pain and regulate the sensitivity of visceral afferents in rats suffering from colitis (Matayoshi et al., 2005; Qiao et al., 2008). With systemic stress, NPY is released, regulates endocrine, behavior, stress, anxiety, appetite, and circadian rhythms, and functions in defecation and food intake (Adrian et al., 1983; Zhou et al., 2008). The level of NPY is increased in the hypothalamus and serum of mice suffering from colitis (Hassan et al., 2014; Reichmann et al., 2015). NGF is a nutrient protein for nerve cells and promotes the repair of damaged nerve fibers (Bilderback et al., 1999). At the site of inflammation, NGF concentration is increased, and cytokines promote the synthesis of NGF by neurons and other cells such as epithelial and endothelial cells (März et al., 1999; Minnone et al., 2017). In this study, concentrations of these neuropeptides were increased in serum and small intestine of rats in the LPS group. The increased levels were reduced with PQQ treatment. The optional PQQ dose approved to be 5.0 mg/kg b.w. The concentrations of IL-1β and TNF-α display a similar pattern to neuropeptides, suggesting that neuropeptides regulated immune factors concentrations. In the colon, SP and VIP concentrations showed the same trends as observed in the jejunum. This might be due to the main site of inflammation challenged with LPS. Additionally, P and VIP play the main role of immunomodulators in the nervous system. Treatments with PQQ increased concentrations of BDNF, NPY, and NGF in the colon which might be related to their neurotropic effects and the damage caused by the LPS challenge. These neuropeptides function in stress and neurotropic. When the intestine is damaged, the neuropeptides secreted by the ENS aim to promote neuronal survival and maintain normal functions (Holzer, 1998; Arciszewski et al., 2008; Qiao et al., 2008). We deduced that PQQ played a different role in different pathological states, and we would investigate further. In conclusion, the results reported in this study suggest that PQQ influenced the release of neuropeptides which regulated the small intestine. We conclude that PQQ regulated enteric neurochemical plasticity of SP-, CGRP-, VIP-, BDNF-, NPY-, and NGF-immunoreactive neurons of weaned rats.

Expression of p-Akt decreased in jejunum and colon with PQQ supplementation. Akt can be phosphorylated by PI3K. Activated Akt can reduce and improve the activity of glycogen synthase kinase-3β (GSK-3β) by phosphorylation of Ser9 and Tyr216 sites, respectively (Majewska and Szeliga, 2017). GSK-3β is associated with cell survival and apoptosis, which can promote peripheral nerve regeneration, improve regrowth of synapses after peripheral nerve damage, and play a role in neurodegenerative diseases (Kitagishi et al., 2014; Huang et al., 2017). Activated GSK-3β can induce Tau perphosphate. Tau protein, a microtube-related protein, mainly acts on the far end of the axon, participates in axon transport, and interacts with the microtube protein, Tubulin, to stabilize the microtube and regulate NMDA receptor signaling pathways (Goedert et al., 1996; Iqbal et al., 2016; Falcon et al., 2019). By regulating GSK-3β and Tau protein, the PI3K/Akt signaling pathway affects a variety of CNS diseases, such as Alzheimer’s disease, Parkinson’s disease, and Huntington’s disease (Kitagishi et al., 2014; Rai et al., 2019). PQQ inhibits apoptosis in the rat hippocampus by regulating the Akt/GSK-3β pathway (Zhou et al., 2020). In addition, PQQ can regulate memory *via* maintaining activation of GSK-3β while reducing the expression of p-AKT (Zhou X. Q. et al., 2018). In this study, PQQ reduced the magnitude of increased p-Akt in jejunum and colon of rats caused by LPS treatment which suggests that PQQ might regulate the secretory functions and structure of the ENS *via* the Akt signaling pathway.

In conclusion, based on ADG, jejunal morphology, immune responses, and enteric neuropeptide expression, we conclude that the intestinal health of weaned rats was damaged by the LPS challenge. Dietary PQQ supplementation reduced inflammatory injury and regulated neurochemical plasticity *via* the Akt signaling pathway in the intestine of rats suffering from enteritis.

## Data Availability Statement

The original contributions presented in the study are included in the article/Supplementary Material, further inquiries can be directed to the corresponding author.

## Ethics Statement

The animal study was reviewed and approved by the Laboratory Animal Welfare and Animal Experimental Ethical Inspection Committee of China Agricultural University (AW01211202-1-2).

## Author Contributions

CS: conceptualization, data curation, formal analysis, investigation, methodology, and writing—original draft. SX, CH, ZW, WW, DM, XY, and HL: data curation and methodology. FW: conceptualization, writing – review, supervision, and funding acquisition. All authors have read and agreed to the published version of the manuscript.

## Conflict of Interest

The authors declare that the research was conducted in the absence of any commercial or financial relationships that could be construed as a potential conflict of interest.

## Publisher’s Note

All claims expressed in this article are solely those of the authors and do not necessarily represent those of their affiliated organizations, or those of the publisher, the editors and the reviewers. Any product that may be evaluated in this article, or claim that may be made by its manufacturer, is not guaranteed or endorsed by the publisher.
