# Supplementary material for: Pyrroloquinoline Quinone Regulates Enteric Neurochemical Plasticity of Weaned Rats Challenged With Lipopolysaccharide
Source: Front Neurosci. 2022 May 3;16:878541. doi: 10.3389/fnins.2022.878541 (PMC9112857; doi:10.3389/fnins.2022.878541)
Supplement: Supplementary file 1 [file Table_1.docx]

Supplementary Material

# Supplementary Figures and Tables

## Supplementary Figures

**
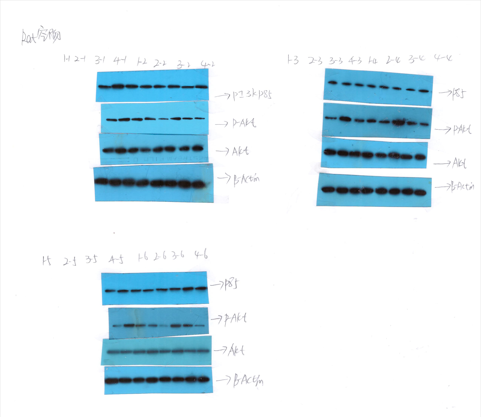
**

**Supplementary Figure 1.** Original scanned image of the bands for p85, p-AKT, AKT and 𝛽-actin in jejunal tissues of rats (n=6).

**
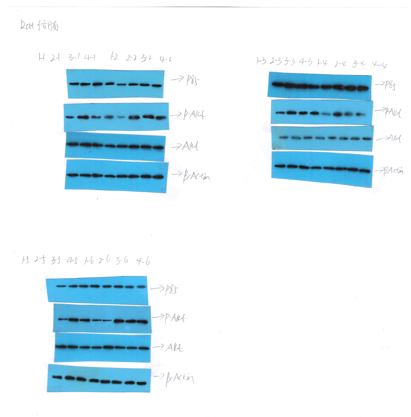
**

**Supplementary Figure 2.** Original scanned image of the bands for p85, p-AKT, AKT and 𝛽-actin in colonic tissues of rats (n=6).

## Supplementary Tables

**Supplementary Table 1.** Primers used for qRT-PCR^1^

| Genes | Primer sequence (5′ - 3′) | Product size, bp | GenBank No. |
| --- | --- | --- | --- |
| SP | F:TGGGCAACGTAGTGGTGATA | 144 | NM_012667.3 |
|  | R:TGTGGACTGCGTAGGTGAAG |  |  |
| CGRP | F:GAACTTGAACGCCATCACCT | 173 | NM_053670.3 |
|  | R:GGATCTCAACAGCGGTCATT |  |  |
| BDNF | F:TGGCTGACACTTTTGAGCAC | 131 | NM_001270638.1 |
|  | R:CAAAGGCACTTGACTGCTGA |  |  |
| NPY | F:TACTCCGCTCTGCGACACTA | 72 | NM_012614.2 |
|  | R:TGTCTCAGGGCTGGATCTCT |  |  |
| NGF | F:CACCACGACTCACACCTTTG | 92 | NM_001277055.1 |
|  | R:CACACACGCAGGCTGTATCT |  |  |
| *β*-actin | F: CTGAGAGGGAAATCGTGCGT | 186 | NM_031144.3 |
|  | R: AGGAAGGCTGGAAAAGAGCC |  |  |

^1^SP, substance P; CGRP, calcitonin gene-related peptide; BDNF, brain-derived neurotrophic factor; NPY, neuropeptide Y; NGF, nerve growth factor.

**Supplementary Table 2.** **Assay kits for ELISA^1^**

| Elisa kits | Company | Catalog number | absorbances |
| --- | --- | --- | --- |
| IL-1β | Beijing Kangjia Hongyuan Biotechnology Co., Ltd | KJEIA0001D | 450 nm |
| IL-6 | Beijing Kangjia Hongyuan Biotechnology Co., Ltd | KJEIA0006D | 450 nm |
| IL-8 | Beijing Kangjia Hongyuan Biotechnology Co., Ltd | KJEIA0008D | 450 nm |
| TNF-α | Beijing Kangjia Hongyuan Biotechnology Co., Ltd | KJEIA0018D | 450 nm |
| SP | Beijing Kangjia Hongyuan Biotechnology Co., Ltd | KJEIA0088 | 450 nm |
| CGRP | Beijing Kangjia Hongyuan Biotechnology Co., Ltd | KJEIA0035D | 450 nm |
| VIP | Beijing Kangjia Hongyuan Biotechnology Co., Ltd | KDEIA0089 | 450 nm |
| BDNF | Beijing Kangjia Hongyuan Biotechnology Co., Ltd | JEN-011B | 450 nm |
| NPY | Phoenix Pharmaceuticals, INC. | # EK-049-03 | 450 nm |
| NGF | Beijing Kangjia Hongyuan Biotechnology Co., Ltd | KJEIA0090 | 450 nm |

^1^SP, substance P; CGRP, calcitonin gene-related peptide; VIP, vasoactive intestinal peptide; BDNF, brain-derived neurotrophic factor; NPY, neuropeptide Y; NGF, nerve growth factor.

**Supplementary Table 3.** Primary antibodies for immunohistochemistry

| Primary antibodies | Company | Catalog number |
| --- | --- | --- |
| UCHL1/PGP9.5 Polyclonal Antibody | Proteintech | 14730-1-AP |
| Anti-Substance P antibody | Abcam | ab14184 |
| CRCP Polyclonal Antibody | Proteintech | 14348-1-AP |
| BDNF Antibody | Affinity | DF6387 |
| NPY Antibody | Affinity | DF6431 |
| NGF Polyclonal Antibody | Immunoway | YT3114 |
